# The Role of Vitamin D in Neuroprotection in Multiple Sclerosis: An Update

**DOI:** 10.3390/nu15132978

**Published:** 2023-06-30

**Authors:** Amarpreet Sangha, Michaela Quon, Gerald Pfeffer, Sarah-Michelle Orton

**Affiliations:** 1 Faculty of Science and Technology, Mount Royal University, Calgary, AB T3E 6K6, Canada; 2 Hotchkiss Brain Institute, Department of Clinical Neurosciences, Cumming School of Medicine, University of Calgary, Calgary, AB T2N 4N1, Canada; 3 Alberta Child Health Research Institute, Department of Medical Genetics, Cumming School of Medicine, University of Calgary, Calgary, AB T2N 4N1, Canada

**Keywords:** vitamin D, multiple sclerosis, neuroprotection, neurodegeneration, 1,25(OH)_2_D_3_

## Abstract

Multiple sclerosis (MS) is a complex neurological condition that involves both inflammatory demyelinating and neurodegenerative components. MS research and treatments have traditionally focused on immunomodulation, with less investigation of neuroprotection, and this holds true for the role of vitamin D in MS. Researchers have already established that vitamin D plays an anti-inflammatory role in modulating the immune system in MS. More recently, researchers have begun investigating the potential neuroprotective role of vitamin D in MS. The active form of vitamin D, 1,25(OH)_2_D_3_, has a range of neuroprotective properties, which may be important in remyelination and/or the prevention of demyelination. The most notable finding relevant to MS is that 1,25(OH)_2_D_3_ promotes stem cell proliferation and drives the differentiation of neural stem cells into oligodendrocytes, which carry out remyelination. In addition, 1,25(OH)_2_D_3_ counteracts neurodegeneration and oxidative stress by suppressing the activation of reactive astrocytes and M1 microglia. 1,25(OH)_2_D_3_ also promotes the expression of various neuroprotective factors, including neurotrophins and antioxidant enzymes. 1,25(OH)_2_D_3_ decreases blood–brain barrier permeability, reducing leukocyte recruitment into the central nervous system. These neuroprotective effects, stimulated by 1,25(OH)_2_D_3_, all enhance neuronal survival. This review summarizes and connects the current evidence supporting the vitamin D-mediated mechanisms of action for neuroprotection in MS.

## 1. Introduction

Multiple sclerosis (MS) is a demyelinating autoimmune disease of the central nervous system (CNS), characterized by an interplay of genetic and environmental factors [1]. Vitamin D deficiency during childhood and adolescence is a risk factor for the development of MS [2]. Vitamin D is obtained primarily via sun exposure (UVB, wavelengths ~295–315 nm) and/or taking vitamin D supplements, with limited intake from food in most populations. Higher MS prevalence and earlier onset are associated with geographical locations of increasing latitude (away from the equator) and/or with reduced annual sunlight exposure [3,4,5,6,7,8]. High consumption of fatty fish, a select dietary source of vitamin D, is believed to help protect against this latitude gradient MS association [9,10,11]. Furthermore, evidence has shown that HLA-DRB1*1501, the strongest genetic association in MS, is regulated by 1,25-dihydroxyvitamin D3 (1,25(OH)_2_D_3_) via a vitamin D response element [12,13,14].

Arguably, the strongest evidence comes from large longitudinal population-based cohorts with banked serum samples from persons with MS (pwMS) at time points prior to their MS onset. Using samples from large prospective cohorts of nurses and military personnel in the US, Munger and colleagues found a significant reduction in MS risk (30–60%) associated with the highest quintile of serum 25-hydroxyvitamin D3 (25(OH)D_3_) [15,16]. Scandinavian registry cohorts observed similar results using banked maternal and newborn serum samples—lower circulating 25(OH)D_3_ was associated with significant increases in MS risk [17,18].

With regards to altering disease activity after MS diagnosis, some studies have shown that vitamin D supplementation can reduce relapses and MRI lesion activity in pwMS [19,20,21,22,23,24]. Randomized controlled trials are difficult to execute due to the requirements of large sample size and study length, disease heterogeneity, the sensitivity of detecting differences in clinical endpoints, retention rates, the influence of sun exposure, and vitamin D maintenance dose in the control arms, among other challenges. Several studies have shown promising results with respect to certain clinical endpoints with high-dose vitamin D supplementation compared to placebo groups [25,26,27,28,29]. However, consistent results as to the long-term clinical benefits of MS are lacking. It may be likely that RCTs with vitamin D supplementation will never be able to assess its potential benefits, especially given that a control arm of zero supplementation is deemed non-ethical [30].

Both the circulating and biologically active forms of vitamin D (25(OH)D_3_ and 1,25(OH)_2_D_3_, respectively) cross the blood–brain barrier (BBB) into the CNS, where they can act on various neuronal and glial cells [31,32]. Neurons, microglia, and astrocytes express 1α-hydroxylase (CYP27B1), the enzyme responsible for converting 25(OH)D_3_ into 1,25(OH)_2_D_3_ [33,34,35,36]. Along with oligodendrocytes, these cells also all express the vitamin D receptor (VDR) [33,34,35,36,37,38,39]. 1,25(OH)_2_D_3_ can thus be synthesized in the cytosol or diffuse through the plasma membrane of target cells and bind to the VDR in the cytoplasm [40]. The VDR-1,25(OH)_2_D_3_ complex then translocates to the nucleus where it couples with the retinoid X receptor (RXR) and binds to the vitamin D response elements of target genes to regulate their expression [40]. This leads to the biological actions of vitamin D, mediated through changes in the expression of a huge array of target genes, involved in diverse functions ranging from bone health to CNS development to immunomodulation.

Both inflammatory demyelinating and subsequent neurodegenerative processes contribute to the complex pathogenesis of MS [41]. The autoimmune component is driven by the T-cell-mediated attack of the myelin sheath surrounding the axons of CNS neurons, which prevents efficient impulse transmission, leading to neurological symptoms [42,43]. These abnormal immune responses in MS cause focal inflammatory demyelinated lesions in the CNS, and chronic myelin destruction enhances axonal damage and eventual neuronal loss [41,44,45,46]. Remyelination is important in preventing permanent disability, but the ability to restore myelin becomes increasingly impaired with progressive MS [47]. Neuroprotective treatment strategies—aimed at preventing initial loss or promoting remyelination—remain elusive in MS treatment [46,47]. Understanding the mechanisms by which vitamin D deficiency affects immune regulation, myelination, and neurodegeneration is important to potentially altering MS risk and disease activity.

The mechanism of vitamin D-mediated immunological activity, including reducing inflammation, is much more established in the literature than its proposed neuroprotective benefits [48,49,50]. Evidence that vitamin D sufficiency is important in combatting axonal degeneration, as well as both glial and neuronal loss in MS, has been rather limited in the past [48,50]. However, the number of studies investigating the neuroprotective mechanisms of vitamin D is growing. As such, the intent of this review is to examine the current state of research in order to summarize and clarify the latest findings regarding the mechanistic role of vitamin D-mediated neuroprotection in MS. We map out its actions via the following mechanisms: enhancing oligodendrocyte lineage differentiation, enhancing neurotrophin expression, attenuating aberrant microglial and reactive astrocyte activation, stabilizing the BBB, and reducing oxidative stress.

## 2. Promoting Oligodendrocyte Proliferation and Differentiation

Oligodendrocyte dystrophy and apoptosis are significant pathological features in the demyelinating lesions of MS [51]. Oligodendrocytes are myelin-producing glial cells that support neurons in the CNS. During periods of tissue injury, mature oligodendrocytes have the ability to remyelinate CNS neuronal axons to maintain saltatory conduction, which is a prerequisite for proper brain functioning [52]. Remyelination by oligodendrocytes can be robust and restorative, especially in early MS, but declines during later stages of the disease [52,53]. The ability to regenerate the oligodendrocyte population depends on the availability of neural stem cells (NSCs) and oligodendrocyte progenitor cells (OPCs) [52]. Oligodendrogenesis is the process by which NSCs commit to an oligodendrocyte lineage and differentiate into OPCs, which ultimately differentiate into oligodendrocytes [54]. However, in MS, especially during the progressive stage, the regenerative capacity of NSCs and OPCs to give rise to oligodendrocytes is considerably diminished, contributing to neuronal degeneration and impaired axonal conduction [6,53,55].

It has previously been shown that OPCs and oligodendrocytes express VDR [34]. VDR-RXR heterodimerization is present in OPCs and is necessary for OPC differentiation [37,56]. The capacity of 1,25(OH)_2_D_3_ to promote OPC differentiation is diminished in the presence of a VDR antagonist in a dose-dependent manner [37]. By blocking VDR, it becomes apparent that 1,25(OH)_2_D_3_ is exerting its proposed neuroprotective effect on oligodendrocyte lineage cells via VDR-RXR signalling [37]. In 2015, it was demonstrated that VDR is constitutively expressed in NSCs [57]. Increasing vitamin D in vitro upregulates VDR expression in NSCs in a dose-dependent manner [57].

**Figure 1:**
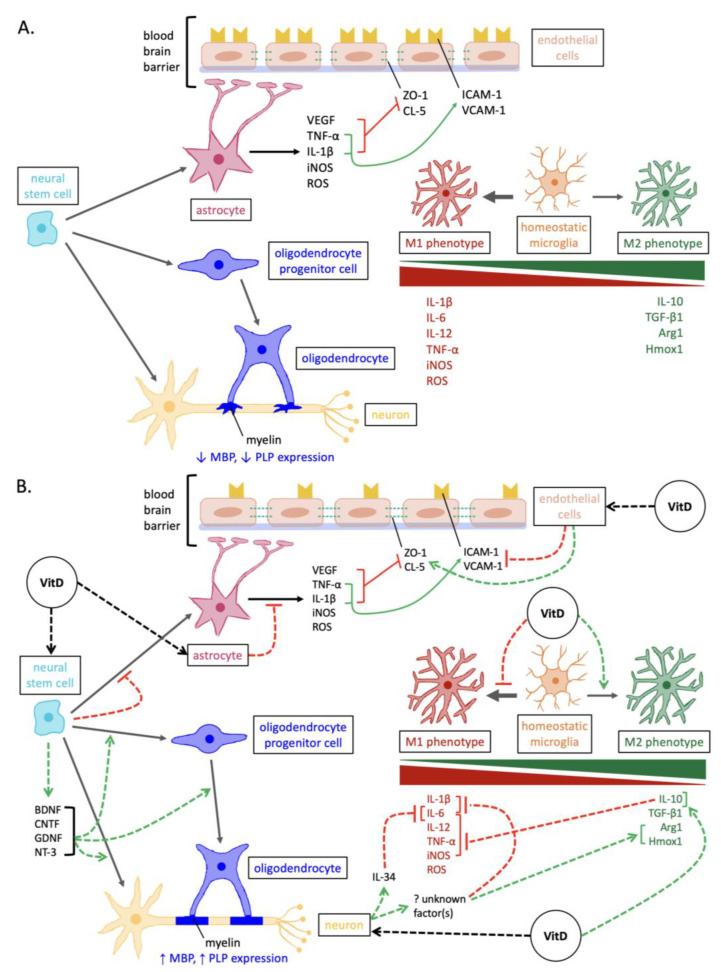
Overview of the mechanisms involved in neurodegeneration versus vitamin D-mediated neuroprotection in MS. (**A**) Pathways of neurodegenerative pathogenesis in MS. In addition to the depicted, the expression of neurotrophins and antioxidant enzymes is reduced in neurons and glia. (**B**) Neuroprotective pathways elicited by vitamin D in MS. Abbreviations: Arg1, arginase 1; BDNF, brain-derived neurotrophic factor; CNTF, ciliary neurotrophic factor; CL-5, claudin-5; GDNF, glial cell line-derived neurotrophic factor; Hmox1, heme oxygenase 1; ICAM-1, intercellular cell adhesion molecule-1; iNOS, inducible nitric oxide synthase; IL, interleukins; MBP, myelin basic protein; NT-3, neurotrophin-3; PLP, proteolipid protein; ROS, reactive oxygen species; VCAM-1, vascular cell adhesion molecule-1; TGF, transforming growth factor; TNF, tumor necrosis factor; VEGF, vascular endothelial growth factor; ZO-1, zonula occluden-1.

Increased 1,25(OH)_2_D_3_ exposure stimulates an increase in NSC proliferation and, importantly, increases the proportion of NSCs that can differentiate into oligodendrocyte lineage cells [57,58] (Figure 1). In a study utilizing a lysolecithin-induced model in the corpus callosum of male rats, the group that received oral 1,25(OH)_2_D_3_ had a higher concentration of OPCs at lesion sites compared to sham and control groups [58]. Furthermore, 1,25(OH)_2_D_3_ administration increases the proportion of mature oligodendrocytes in a cuprizone-induced demyelination model as well as in NSC and OPC cultures [37,57,59]. Consistent results were found in a murine experimental autoimmune encephalomyelitis (EAE) model, where 1,25(OH)_2_D_3_ administration increased the concentration of NSCs, OPCs, and oligodendrocytes [60]. EAE models are often considered more reflective of MS pathogenesis than demyelination models as they exhibit both immune-mediated inflammation and demyelination [61]. Additionally, 1,25(OH)_2_D_3_ administration upregulates the expression of myelin basic protein and proteolipid protein, which are markers of myelin content [37,58,59,60]. The upregulation of these markers may suggest that demyelination is reduced and/or remyelination is increased in response to 1,25(OH)_2_D_3_ [37,58,59,60].

## 3. Enhancing Neurotrophin Expression

Reduced neurotrophin secretion is another factor that contributes to inadequate neuroprotection in neurodegenerative disorders such as MS [62,63]. Neurotrophins are a family of proteins that elicit protective and regenerative effects by stimulating the proliferation and differentiation of NSCs, as well as the growth, survival, and proper functioning of neuronal and glial cells [62,64,65]. Neurotrophins are secreted by multiple cell types, some of which include NSCs, neurons, oligodendrocytes, astrocytes, and M2 microglia [57,65,66,67,68]. Key neurotrophins include NT-3, BDNF, CNTF, GDNF, and NGF [64].

Vitamin D has previously been demonstrated to increase neurotrophin expression [69,70,71]. When mouse NSCs were cultured with 1,25(OH)_2_D_3_, the expression of NT-3, BDNF, CNTF, and GDNF was upregulated [57]. Consistent results were observed in rodent models showing the upregulation of NGF and BDNF in CNS tissue following 25(OH)D_3_ supplementation [71,72,73]. In addition, 1,25(OH)_2_D_3_ exposure stimulated oligodendrogenesis and neurogenesis in mouse NSCs [57]. This effect may be mediated by the induction of these neurotrophins, whereby 1,25(OH)_2_D_3_ enhances NSC proliferation and differentiation into neurons and oligodendrocytes [57]. These neurotrophins have all been previously associated with enhanced oligodendrogenesis and neurogenesis [74,75,76,77,78,79,80]. Overall, increased neurotrophin secretion in response to vitamin D may tip the balance towards a less neurotoxic environment in which CNS cells can more effectively contribute to repair and regeneration (Figure 1).

## 4. Attenuating the Activation of Pro-Inflammatory/Neurodegenerative Microglia

Microglia play a central role in normal CNS development and maintenance, including resident immune surveillance. In MS, activated microglia are abundant in the focal plaques of demyelination and contribute to disease progression [81,82]. The polarization of activated microglia has typically been classified into two opposing phenotypes, which likely exist on a continuum: M1 microglia, which are considered pro-inflammatory and neurotoxic, or M2 microglia, which are considered anti-inflammatory and neuroregenerative [66,81,83]. The classically activated M1 phenotype contributes to neurodegeneration via the release of reactive oxygen species (ROS) and pro-inflammatory cytokines, as well as enhancing antigen presentation to CD4+ T cells [66,67,81,83,84,85].

There are various lines of evidence supporting the attenuation of the M1 phenotype with increased vitamin D exposure [35,39,58,59,86,87,88,89]. Firstly, the release of pro-inflammatory cytokines TNF-α, IL-1β, IL-6, and IL-12, as well as inducible nitric oxide synthase (iNOS), ROS formation, and CD86 (a marker of the M1 phenotype) are reduced in cultured microglia exposed to 1,25(OH)_2_D_3_ and in vitamin D-supplemented mouse models of CNS disease [35,86,88,89,90,91,92]. A reduction in these M1 cytokines and ROS then contributes to oligodendrocyte and neuronal survival [67,93,94,95,96,97]. The vitamin D-mediated phenotypic shift also downregulates MHC II expression by M1 microglia, reducing their antigen-presenting capacity to CD4+ T cells [39,84,88,96], which fosters an environment that permits tissue repair.

Vitamin D helps mediate a microglial shift towards the M2 phenotype [98]. The alternative M2 phenotype is neuroprotective in nature, given its association with the increased secretion of anti-inflammatory cytokines as well as the upregulation of neurotrophins and ROS-regulating enzymes arginase 1 and heme oxygenase 1 [91,99]. In a mouse model of Parkinson’s disease, vitamin D has been shown to increase the expression of CD163, CD206, and CD204, which are all markers of M2 microglia [90]. Furthermore, several studies have demonstrated that vitamin D is associated with increased concentrations of M2-associated cytokines, IL-4, IL-10, and TGF-β1, in both EAE mice and in serum from pwMS [100,101,102,103]. These cytokines are implicated in regenerative CNS functions, such as oligodendrogenesis and neurogenesis [104,105,106]. In addition, M2 microglia have a stronger phagocytic capacity to engulf myelin debris, which is a prerequisite for myelin repair in CNS disorders (due to the inflammatory and neurotoxic nature of myelin debris) [39,66,67,83,107]. Given that 1,25(OH)_2_D_3_ helps to promote a shift towards the M2 microglial phenotype [88,108,109], this could potentially also enhance the clearance of myelin debris.

The vitamin D-induced release of IL-10 from microglia is one mechanism mediating the M1 to M2 phenotypic shift [35]. Boontanrart et al. (2016) exposed cultured microglia to LPS, IFN-γ, and Theiler’s murine encephalomyelitis virus to induce M1 polarization [35]. In these M1 microglial cultures, they found that 1,25(OH)_2_D_3_ stimulated microglia to release IL-10, which binds to the microglial IL-10 receptor in an autocrine and paracrine manner to upregulate SOCS3 [35]. SOCS3 then acts via a negative feedback loop to downregulate TNF-α, IL-6, IL-12, and iNOS [35].

Another mechanism by which vitamin D is hypothesized to shift microglial phenotypes is via its stimulation of neuronal factors such as IL-34 [39]. In vitro experiments were conducted on neurons and LPS-activated M1 microglia, revealing that 1,25(OH)_2_D_3_ stimulates neurons to release factor(s) that act on microglia to influence a transition from M1 to M2 polarization, as evidenced by downregulated IL-6, IL-1β, and MHC II, as well as upregulated heme oxygenase 1 and arginase 1 [39]. Neuronal IL-34, in particular, is a survival factor, contains a VDRE in its promoter region, and its expression is slightly increased when stimulated by 1,25(OH)_2_D_3_ [39,97]. Under the influence of 1,25(OH)_2_D_3_, neuronal IL-34 inhibited the expression of IL-6 in M1 microglia, partially contributing to the phenotypic transition from M1 to M2 polarization [39]. This indicates that other unknown neuronal factor(s) are likely involved in facilitating the microglial shift towards the M2 phenotype under the influence of 1,25(OH)_2_D_3_ [39]. Overall, it is hypothesized that vitamin D promotes a shift away from M1 and towards the M2 microglial phenotype, thus reducing damage to neurons and oligodendrocytes, promoting greater potential for repair and recovery (Figure 1).

## 5. Reducing Reactive Astrogliosis

Astrocytes comprise a large portion of the glial cell population in the CNS [110]. They elicit essential functions, such as metabolically assisting neuronal growth, signalling immune cell entry into the CNS, and forming a critical component of the BBB [110]. When CNS injury occurs, astrocytes become reactive and divide rapidly, also termed astrogliosis, which has both positive and negative consequences [110,111]. Reactive astrocytes aid in recovery by encompassing the site of demyelination, resulting in the construction of a glial scar, which prevents the injury from expanding [112]. However, after a certain point, the abnormal increase in the number of reactive astrocytes is detrimental, as it contributes to the development of MS lesions [110,113,114]. Reactive astrocytes release a number of pro-inflammatory cytokines and ROS, which can be neurotoxic to OPCs, oligodendrocytes, and neurons [115].

Reducing the activation and abundance of astrocytes may make the neurodegenerative microenvironment more conducive to repair processes [67,116]. MS plaques with fewer reactive astrocytes exhibit elevated OPC content and greater remyelination [116]. The expression of VDR and CYP27B1 were upregulated in the astrocytes of LPS-stimulated rats, supporting a potential response via vitamin D [36]. In rodent models of cuprizone-induced demyelination and LPS injection, it was shown that the concentration and activation of astrocytes were decreased in mice that were administered intraperitoneal injections of 25(OH)D_3_ and 1,25(OH)_2_D_3_ [36,59]. Findings from other rodent CNS disease models have similarly supported a decrease in GFAP expression and astrocyte activation upon supplementation with oral or injected vitamin D [36,71]. More specifically, 25(OH)D_3_ and 1,25(OH)_2_D_3_ downregulated iNOS, TLR4, TNF-α, and IL-1β in cultured astrocytes and EAE [36,117,118] (Figure 1). Additionally, the in vitro exposure of mouse NSCs to 1,25(OH)_2_D_3_ reduces NSC differentiation into astrocytes [57] (Figure 1). This is interesting, as vitamin D has the opposite effect of increasing NSC differentiation into oligodendrocytes and neurons (discussed above) [57], consistent with its role in neuroprotection.

## 6. Stabilizing the Blood–Brain Barrier

The BBB regulates the movement of blood-borne molecules, ions, and cells into the CNS, leading to the stabilization and protection of the neuronal microenvironment [119,120,121,122]. Breakdown of the BBB and consequent hyperpermeability occurs early in MS [123]. When stimulated by pro-inflammatory cytokines from various immune cells, endothelial cells of the BBB downregulate tight junctions and upregulate cell-adhesion molecules, which destabilizes the BBB and increases leukocyte recruitment into the CNS, respectively [124]. Reactive astrocytes also play a role in BBB instability [120,125,126]. Pro-inflammatory cytokines, including TNF-α and IL-1β, secreted from reactive astrocytes stimulate the endothelial cells to downregulate tight junctions and upregulate cell-adhesion molecules [120,125,126]. The reactive astrocytes also detach their endfeet processes from the capillary endothelium, making the BBB more permeable [127]. Interestingly, in a neurodegenerative environment, reactive astrocytes release vascular endothelial growth factor (VEGF), which signals endothelial cells to lower tight junction expression, which destabilizes the BBB [128]. As a result of BBB hyperpermeability, CD4+ Th1 and Th17 cells are able to translocate into the CNS, where their secreted cytokines prompt the degeneration of oligodendrocytes and myelinated axons [123].

Vitamin D is thought to counteract BBB hyperpermeability through multiple mechanisms (Figure 1). In a study using human brain endothelial cells, the effects of 1,25(OH)_2_D_3_ exposure were examined following exposure to TNF-α and exposure to sera derived from MS patients [129]. It was found that 1,25(OH)_2_D_3_ can act directly on endothelial cells to upregulate tight junction proteins (zonula occluden-1 and claudin-5) and downregulate cell adhesion molecules (ICAM-1 and VCAM-1) [129]. These two outcomes both contribute to BBB stabilization [129]. More recently, de Oliveira et al. (2020) reported similar findings, observing that 1,25(OH)_2_D_3_ supplementation in EAE mice upregulated zonula occluden-1 and lowered BBB permeability, alongside symptom improvement [88]. They also analyzed immune cell entry into the CNS and axonal loss; 1,25(OH)_2_D_3_-stimulated BBB stabilization was associated with the limited migration of immune cells into the CNS and reduced demyelination scores [88]. Additionally, reactive astrocytes exposed to 25(OH)D_3_ demonstrate decreased expression of TNF-α, IL-1β, and VEGF, which may help further stabilize the BBB [36,120,125,126,128] to promote neuroprotection.

In addition to reducing BBB permeability by upregulating tight junction proteins and downregulating cell-adhesion molecules, 1,25(OH)_2_D_3_ has been shown to lower the expression of matrix metalloproteinase-9 (MMP-9) in mouse-brain endothelial cells and in a rat model of ischemic stroke [130,131]. Various cell types, including endothelial cells, CNS cells, and leukocytes, release MMPs [132]. MMPs are responsible for breaking down extracellular matrix components (such as collagen, fibronectin, and laminin) and tight junction proteins, thereby contributing to BBB instability [132,133,134,135]. As such, reducing MMP-9 expression may be another underlying mechanism by which vitamin D promotes BBB stabilization [98]. It has also been demonstrated that 1,25(OH)_2_D_3_ reduces the apoptosis of human endothelial cells exposed to MS sera, which may indicate a further protective effect of vitamin D on the BBB [98,136].

## 7. Reducing Oxidative Stress

Another significant contributor to the pathogenesis of MS is oxidative stress [137]. Normally, ROS, such as hydrogen peroxide, nitric oxide, and superoxide, are generated during cellular respiration when a small quantity of electrons “leak out” of the electron transport chain and react with oxygen [137,138]. Under physiological conditions, ROS that are produced in low amounts are neutralized by antioxidant enzymes present in cells [137]. However, when ROS production exceeds neutralization, the excess ROS can oxidize nucleic acids, proteins, lipids, and carbohydrates, contributing to cell injury and death [137,139]. In MS, the inflammatory environment continually activates a number of cells, including peripheral immune cells, microglia, and astrocytes, that release ROS and pro-inflammatory cytokines, further enhancing inflammation in a positive feedback loop [137]. Microglia and peripheral macrophages are the greatest generators of ROS in MS [96,137]. While ROS production is increased in MS, ROS neutralization is decreased due to lower antioxidant enzyme expression [140,141]. In the CNS microenvironment, excessive ROS are particularly damaging to oligodendrocyte lineage cells and neurons, as these cell types do not have sufficient antioxidant enzymes to neutralize the ROS [96,97,142]. Injuring OPCs and neurons can impair remyelination, leading to neurodegeneration [143].

Vitamin D has been shown to mitigate oxidative stress in the CNS tissue of EAE mice [88]. 1,25(OH)_2_D_3_ treatment reduced the biomarkers of oxidative stress (lipid hydroxides and protein carbonyls), while the expression of antioxidant enzymes (glutathione peroxidase, superoxide dismutase, and catalase) was restored to normal levels [85]. Similarly, vitamin D sufficiency and supplementation have been associated with decreased oxidative stress and increased antioxidant biomarkers in both animal models [144,145,146,147] and human studies [148,149,150,151,152] of other health conditions, including Type II Diabetes. In addition, as discussed, vitamin D helps decrease the M1 microglial population, a potent contributor to elevated nitric oxide and reactive oxygen intermediates in MS. 1,25(OH)_2_D_3_ can downregulate iNOS in activated microglia in culture and in reactive astrocytes from EAE mice [35,118]. In contrast, 1,25(OH)_2_D_3_ may promote the ability to neutralize ROS in microglia by upregulating the expression of heme oxygenase 1 and arginase 1 [39].

Additionally, Nrf2 is an intracellular factor that helps protect cells against oxidative stress by inducing the transcription of various antioxidant enzymes [50,153]. It has been found that the induction of EAE in a mouse model progresses more quickly and more severely in Nrf2 knockout mice compared to wild-type mice [154]. iNOS levels were also significantly increased in the Nrf2 knockout mice versus the wild-type mice [154]. Nrf2 expression is high in the active MS lesions, especially in the neurons and glia, of post-mortem brain tissue in pwMS [155]. 1,25(OH)_2_D_3_ has been demonstrated to increase the expression of Nrf2 alongside heme oxygenase-1 and NAD(P)H quinone oxidoreductase-1 in the CNS tissue of a neurodegenerative mouse model [153]. It has been hypothesized that 1,25(OH)_2_D_3_ simulates the activation of Nrf2, after which Nrf2 translocates into the nucleus to bind to the antioxidant response element to upregulate antioxidant enzyme expression [50,153]. In summary, vitamin D-mediated antioxidant synthesis and reduced ROS production provide a path by which CNS cells reduce oxidative stress to promote neuroprotection (Figure 1).

## 8. Conclusions

MS continues to have a large impact worldwide, with an estimated 2.9 million people living with MS [156]. There are two key intertwined facets of MS pathophysiology: inflammation and neurodegeneration. The bulk of MS research and therapeutic targets focus primarily on immunomodulation, while research on how to combat the neurodegenerative components of MS remains much more limited. Neuroprotective strategies continue to represent a promising area of research that could yield strategies to directly target specific cell types and components of the nervous system. Intentional neuroprotective strategies, either on their own or in conjunction with immunomodulatory therapies, may be effective in attenuating the neurodegenerative processes in MS [2,45].

Vitamin D is a proposed neuroprotective agent in MS. We summarized a number of recent studies that investigated the neuroprotective effects of vitamin D, reporting favourable results [36,37,39,57,58,59,60,85,89]. Overall, vitamin D supports the neuronal population in various ways. Under the influence of vitamin D, NSCs express various neurotrophins, namely NT-3, BDNF, CNTF, and GDNF [57]. These neurotrophins then stimulate neurogenesis and protect neurons from degeneration and apoptosis [57,64]. In addition to increasing neurotrophin expression, vitamin D helps promote a shift in the microglial population towards the M2-like phenotype, which is associated with the secretion of anti-inflammatory cytokines to help counteract neuronal damage [35,100,101,102,103]. Vitamin D also promotes neuronal survival by suppressing M1 microglia and reactive astrocytes, thus decreasing the secretion of pro-inflammatory cytokines and ROS, sparing neurons from harm [35,36,39,58,59,87,88,89]. In addition, vitamin D helps preserve the integrity and promotes the stabilization of the BBB, decreasing the entry of autoreactive T-cells with the potential to target neurons. Collectively, we see that evidence supports that vitamin D acts via various pathways, implicating a variety of CNS cell types, to promote neuronal integrity and survival.

Combined, these neuroprotective effects elicited by vitamin D promote a more stable microenvironment in which CNS glial cells can more easily participate in repair and recovery processes to help restore the structure and functioning of neurons. One limitation of the proposed mechanisms is that much of the supporting evidence comes from animal models, and potential differences in neuroprotective pathways in humans remain to be discovered [46,82,157]. Future directions for research include extending this work in cultured human neuronal and glial cells, with a potential for some of the findings to be studied in post-mortem brain tissue. With respect to prospective studies employing vitamin D supplementation, assessing endpoints that better capture neuroprotective benefits would be a useful avenue to explore.

This review has connected and mapped out current evidence to summarize the proposed mechanisms of vitamin D neuroprotection in MS. The aim is to promote a better understanding of the potential interactions between CNS cell types stimulated by vitamin D, neuroprotection in MS, and overall outcomes. The combined evidence further suggests that vitamin D supplementation and promoting vitamin D sufficiency at a population level, alongside the development of new neuroprotective agents, remains a worthwhile pursuit in the fight against MS.

## Data Availability

Not applicable.
